# Photocatalysis

**DOI:** 10.3762/bjnano.5.119

**Published:** 2014-07-16

**Authors:** Rong Xu

**Affiliations:** 1 School of Chemical & Biomedical Engineering, Nanyang Technological University, 62 Nanyang Drive, Singapore 637459

**Keywords:** photocatalysis

Finding long-term solutions to meet the growing energy demands of the human society is one of the greatest challenges of our age. Photocatalysis, a topic of many decades of attention, has recently received renewed and more intense interest in developing innovative solutions towards achieving our sustainability goal. Though immensely inspired by natural photosynthesis, the research on artificial photosynthesis is still in its early stage, and many technological challenges must be solved before it can be applied to large-scale.

It has been widely recognized that it is necessary to develop advanced materials and new molecules assembled preferably from earth abundant elements as efficient photocatalysts to accomplish the complex process of solar energy driven water splitting and carbon dioxide reduction. Nanotechnology certainly plays a pivotal role in enabling a rational design of the structures, interfaces and surfaces with controllable features at a length scale comparable to chemical reactions. In this Thematic Series, two review articles present an excellent overview of the significance of nanostructures in visible light photocatalysis in a timely manner.

Many materials aspects of photocatalysts influence the photocatalytic performance, such as the electronic, structural, and morphological features of the semiconductors, and the interface properties between semiconductors and cocatalysts. This Thematic Series contributes to bringing together the research at the frontiers of materials science and nanotechnology to address some of these aspects. It is evidenced from several reports that structural, compositional and morphological tuning, in particular for hybrid materials systems such as Ag–ZnO, VTi/MCM-41, are important toward achieving higher solar energy conversion efficiencies. In a couple of reports, materials alternative to conventional metal oxides, for example, reduced graphene oxide, graphene quantum dots integrated with TiO_2_ nanotube arrays, and carbon nitride, have been explored to construct photocatalysts with enhanced performances.

On the other hand, molecular catalysts have an advantage in design flexibility and structural tunability. A contribution based on the investigation of molecular bismuth vanadium oxide cluster exemplifies these characteristics. Besides solar fuel production, photocatalysis has a long history in water treatment. In this Thematic Series, there is also a report on the latest development in the utilization of mesoporous cerium oxide for visible light-driven dye degradation. In recent years, the interest in photocatalytic organic conversion has risen, which is reflected by a report on photo-epoxidation. And last but not least, one of the studies of this Thematic Series aims for a fundamental understanding of the photophysical process by optical modeling.

I am grateful to all the authors for their valuable contributions. Thanks should also be given to the dedicated Beilstein team for their dependable support.

Rong Xu

Singapore, June 2014

